# To prevent being stressed-out: Allostatic overload and resilience of general practitioners in the era of COVID-19. A cross-sectional observational study

**DOI:** 10.1080/13814788.2021.1982889

**Published:** 2021-10-11

**Authors:** Dóra Békési, Illés Teker, Péter Torzsa, László Kalabay, Sándor Rózsa, Ajándék Eőry

**Affiliations:** a Rácz Károly Clinical Medicine PhD School, Semmelweis University, Budapest, Hungary; b Semmelweis University, Budapest, Hungary; c Department of Family Medicine, Semmelweis University, Budapest, Hungary; d Washington University, St. Louis, USA; e Károli Gáspár University of the Reformed Church, Budapest, Hungary

**Keywords:** Allostatic overload, resilience, general practitioners, COVID-19, recreational resources

## Abstract

**Background:**

Responsibility of general practitioners (GPs) in delivering safe and effective care is always high but during the COVID-19 pandemic they face even growing pressure that might result in unbearable stress load (allostatic overload, AO) leading to disease.

**Objectives:**

We aimed to measure AO of Hungarian GPs during the COVID-19 pandemic and explore their recreational resources to identify potential protective factors against stress load.

**Methods:**

In a mixed-method design, Fava’s clinimetric approach to AO was applied alongside the Psychosocial Index (PSI); Kellner’s symptom questionnaire (SQ) to measure depression, anxiety, hostility and somatisation and the Public Health Surveillance Well-being Scale (PHS-WB) to determine mental, social, and physical well-being. Recreational resources were mapped. Besides Chi-square and Kruskal-Wallis tests, regression analysis was applied to identify explanatory variables of AO.

**Results:**

Data of 228 GPs (68% females) were analysed. Work-related changes caused the biggest challenges leading to AO in 60% of the sample. While female sex (OR: 1.99; CI: 1.06; 3.74, *p* = 0.032) and other life stresses (OR: 1.4; CI: 1.2; 1.6, *p* < 0.001) associated with increased odds of AO, each additional day with 30 min for recreation purposes associated with 20% decreased odds (OR: 0.838; CI: 0.72; 0.97, *p* = 0.020). 3–4 days a week when time was ensured for recreation associated with elevated mental and physical well-being, while 5–7 days associated with lower depressive and anxiety symptoms, somatisation, and hostility.

**Conclusion:**

Under changing circumstances, resilience improvement through increasing time spent on recreation should be emphasised to prevent GPs from the adverse health consequences of stress load.

KEY MESSAGES

- Allostatic overload refers to the dysregulation of stress-related responses leading to disease.
- High-risk and high-gain: the higher the complexity, the higher the potential impact.
- It arises when acute or chronic stress-load exceeds individual coping ability.
- COVID-19 – related allostatic overload caused a huge burden on healthcare professionals, including GPs.
- Active recreation might help staying balanced with elevated well-being.

## Introduction

The ongoing pandemic of COVID-19 turned out to be a strong stressor for all medical doctors, causing psychological distress and mental health problems [1]. It demanded hospitals and specialist care to transform into pandemic centres. This has increased the responsibility of family physicians working in primary care to screen and treat serious cases requiring skills specific to other specialities. They were also expected to run their consultations online without physical examination, and were not sufficiently equipped to contact patients when needed [2]. In the last decades, general practitioners’ physical and mental health has come into focus [3,4]. Besides extreme workload, moral implications for ‘good doctoring’ increased their work-related stress. Major events, but subtle, chronic daily experiences as well – which an individual perceives as stressful – activate regulatory systems (the autonomic, neuroendocrine, metabolic, and immune system) to change a set point and operate at elevated or reduced levels [5,6]. This is called allostasis, the process to achieve stability through change [7]. Increased catecholamine, cytokine and HPA hormone levels are the mediators of this adaptational process resulting in elevated heart rate, blood pressure or inflammation [5]. However, long-term activation of the regulatory systems by repeated stress will lead to overuse and dysregulation of the mediators of allostasis, causing allostatic load, manifesting in anger, fatigue, frustration and feeling out of control (‘stressed-out’) [8]. When challenges exceed the individual’s coping ability, allostatic overload will be the result, a condition with consequent diseases (e.g. hypertension, depression, arthritis, metabolic syndrome or tumorous diseases) [9–11]. To understand the role of allostatic overload in the background of ill-health [12], identification of individual stressors, clinical signs and symptoms directly related to stress sources and the individual’s response to the stressors give the cue [10,13]. Scientific literature concerning GPs’ health focuses primarily on mental ill-health [14]. This is even more essential with the burden of the pandemic on the health care system worldwide. Such an exceptional situation, however, should also lead to exploring sources of resilience beside identifying distress. Increasing well-being will contribute to reaching optimum health through positive affect, personal relationships, and a meaningful and optimistic life [14–16]. Besides, cognitive-behavioural stress-management techniques and mindfulness-based education programmes [14], recreation has recently come into focus as a positive coping response to stress [15,17].

### Study objectives

We targeted to define the prevalence of allostatic overload among Hungarian general practitioners during the first wave of COVID-19 and define the most important factors associated with it. We postulated that the infection and the related confinements and proceeding rules concerning primary health care resulted in significantly increased stress load of professionals. Additionally, we aimed to measure their well-being, regularity, and forms of recreational activity they attain and – consequently – if these might associate with increased mental and physical health or increased resilience against stress load.

## Methods

### Study design and sample recruitment

We performed a voluntary online survey among Hungarian GPs between 28th August and 16th October 2020. Participants were recruited between 28th and 30th August *via* institutional sources (1,262 registered email addresses of surgeries or doctors throughout Hungary) and then one reminder was sent between 8th and 10th September. Our invitation letter contained that the Family Medicine Department at Semmelweis University conducted the survey, the time frame for completing the survey (15–20 min) and we defined our aim as to explore the effects of the previous 6 months (the first wave of the pandemic) on them as family physicians and as persons. We did not offer monetary or non-monetary incentives. Personal data was not collected, but – to allow possible follow up – we generated an ID code for each participant. We constructed our survey so that all answers had to be given to continue with the survey; therefore, participants answered all questions, and we did not need to exclude anyone due to incomplete questionnaire reply.

### Ethics

Online consent was secured by all participants. The study was conducted by the Declaration of Helsinki and was approved by the review board of the Medical Research Council (IV/5657-2/2020/EKU).

### Measurements

*Sociodemographic and health-related characteristics of the sample*. We collected data on participants’ age, gender, and place of living (capital, county seat, town or village); on working conditions (actively working during the pandemic; method of working (personal, phone consultations, other), uncertainty about coronavirus in comparison to the first wave (no change, decreased, increased)). We asked if they took an active role in maintaining their health and the number of days they did recreational activities for at least 30 min. We also asked for the number of chronic diseases, any diagnosed psychiatric disease, the number of prescribed and over-the-counter medicines taken daily.

*Allostatic overload*. We measured COVID-related allostatic overload according to Fava’s definition based on the Diagnostic Criteria for Psychosomatic Research-Revised (DCPR-R) and used the Psychosocial Index (PSI) self-rating questionnaire by the same authors to measure each criterion [13,18,19]. The PSI includes 55 items. Sociodemographic and clinical data are measured from 1 to 12, perceived and objective stress by items 13–20 and 22–30 in a YES/NO format with a maximum score of 17, and well-being by items 31–36 with a score ranging from 0 to 6. Psychological distress is measured by items 37–51 addressing symptoms of sleep disturbances, somatisation, anxiety, depression, and irritability on a 0–3 Likert scale with a maximum score of 45. Abnormal illness behaviour contains items 52–54, concerning bodily preoccupations and hypochondriac beliefs on a 0–3 Likert scale with a range from 0 to 9. Quality of life is measured by one direct question (item 55) with 5 possible choices from excellent to awful [19].

We applied these tools – in accordance with previous research [20,21] – to measure COVID-related allostatic overload (Table 1). Besides measuring individual stressors, our primary focus was on COVID-related allostatic overload. Therefore, we tailored A2 criterion of DCPR-R to COVID as a particular stressor. According to the instructions provided in the DCPR-R allostatic overload is diagnosed when A1 + A2 + B1 or B2 or B3 is present. To measure stress load independent of COVID-19, we applied PSI questions 13–20 and 22–30 [19].

**Table 1.** Clinimetric criteria of allostatic overload based on the Diagnostic Criteria for Psychosomatic Research Revised Semi Structured Interview (DCPR-R-SSI) and the Psychosocial Index (PSI).

Allostatic overload		
	DCPR-R-SSI	PSI
Criterion A	A1	Items
	The presence of a current identifiable source of distress in the form of recent life events and/or chronic stress	Death of a family member Separation from spouse or long-time partner Recent change of job Financial difficulties Moving within the same city Moving to another city Legal problems Beginning of a new relationship Seriously ill close relative
	A2	COVID-specific question
	The stressor is judged to tax or exceed the individual coping skills when its full nature and full circumstances are evaluated	’During the time of the restrictions, did you feel that the changes caused by the coronavirus epidemic were testing or exceeding your capacity?’*
Criterion B	B1	Items
*The stressor is associated with 1 or more of the following 3 features which have occurred within 6 months after the onset of the stressor*	At least two symptoms Difficulty falling asleep Restless sleep Early morning awakening Lack of energy Dizziness Generalised anxiety Irritability Sadness Demoralisation	Long time to fall asleep/restless sleep/waking up too early/feeling tired waking up Stomach, bowel pains Heart beating quickly or strongly without any reason Pressure or tightness in head or body/ dizziness Breathing difficulties Tired, lack of energy Irritable/sad/tense/lost interest Panic attacks
	B2	Items
	Significant impairment in social or occupational functioning	Work-related: satisfying/under pressure/problems with colleagues/unemployed Serious arguments with close relatives/others Tension at home Living alone/feeling lonely Anyone to trust and confide in Getting along well with people
	B3	Items
	Significant impairments in environmental mastery (feeling overwhelmed by the demands of everyday life)	Do you often feel overwhelmed by the demands of everyday life? Do you often feel you cannot make it?

DCPR-R criteria defined allostatic overload with related items from the Psychosocial Index self-rated questionnaire. Text in italics (fulfilling A2 criterion) was formulated to be specific to COVID epidemic as a stressor. PSI does not contain A2 criterion [19,20].

### Mental health and somatisation

Mental health was measured with the Kellner Symptom Questionnaire (SQ) and the Public Health Surveillance Well-being Scale (PHS-WB) [22,23]. SQ consists of four scales: depression, anxiety, somatisation, and hostility, each divided into two subscales, one for the symptoms (depression, anxiety, somatisation and hostility) and the other for well-being (contentment, relaxation, physical well-being and friendliness) [22]. The 10-item shortened version of PHS-WB was used to measure physical, mental, and social well-being. The first five items (on scale 0–5) result in a score of mental well-being. The following two items measure social well-being with scales from 0 to 10. The last three items provide the score of physical well-being after their scales being unified. Total well-being is then calculated from all converted scores [23].

### Qualitative methods

**Figure 1.**
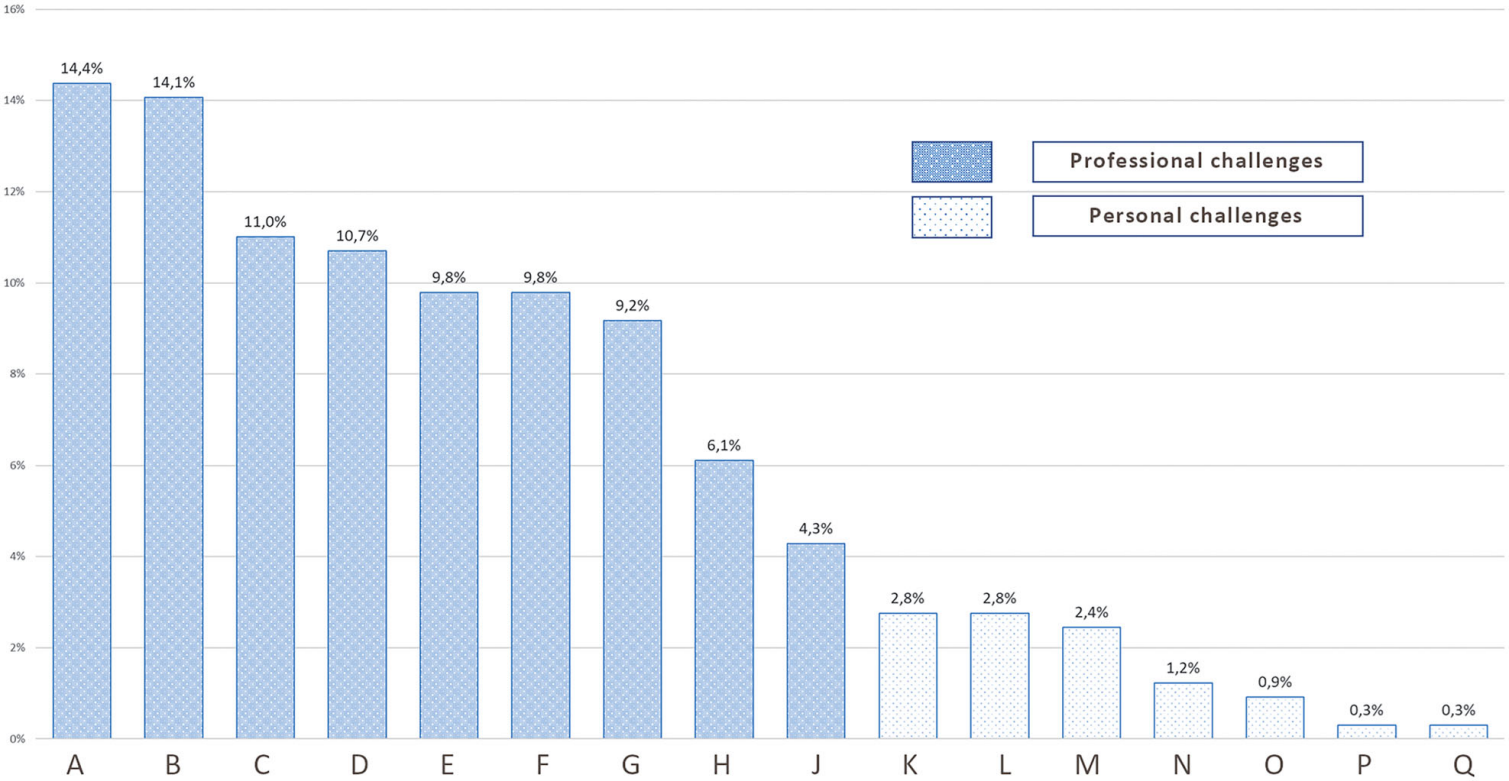
Percentage distribution of professional and personal challenges Hungarian GPs reported related to COVID (*n* = 228). (A) Changes in means of consultation (phone, online), (B) Discontinuation of patient care, patient observations, thus difficulties of diagnosing, (C) Undeveloped proceeding rules and lack of information on them, disorganisation, (D) Increased work-, thus stress load and responsibility due to COVID and unavailability of specialist care, (E) Fear, worry, unreliable information, uncertainty, (F) Panic and worry of patients and to calm and inform them, (G) Lack of protective equipment, (H) Protecting own health, wearing mask, sanitising, (I) Lack of professional contact and help, incompetence of professionals, (J) Lack of personal contact, (K) Increased home workload, organisation, online education, (L) Curfew, travelling restrictions, (M) Opening restrictions, (N) Loss of mental balance, need of psychological help, (O) Financial problems, (P) Loss of loved ones, (Q) Nothing.

To identify the most burdening challenges Hungarian GPs had dealt with in relation to the pandemic, we included the following question in our survey: ‘What was the biggest challenge for you during the epidemic and the quarantine?’ Participants gave free-text answers, which ranged from single-word answers to paragraphs. Following standard qualitative analytical procedures, each researcher read all free-text responses systematically, identified blocks of text that reported factors contributing to allostatic overload, and assigned provisional code names. They compared their coding schemas and agreed on a common one. They then examined the codes, identified themes that organised them into higher-level concepts that explained the origins of overload, constantly comparing their interpretation with the original data, and agreeing on a final interpretation (Tables 2 and 3; Figure 1).

**Table 2.** Categories of COVID-related professional challenges of GPs with description and example responses.

Category	Description of category	Example response
Changes in means of consultation (phone, online)	Responses related to changing proceeding rules to continue consultation with telemedicine	*‘Manage a lot of phone calls and emails’; ‘telephone consultations during physical patient care’*
Discontinuation of patient care, patient observations, thus difficulties of diagnosing	Responses related to lack of personal contact with patients due to online consultations	*‘It was difficult to decide whether there was an urgent and serious condition requiring immediate intervention – based on phone consultation and without physical examination’*
Undeveloped proceeding rules and lack of information on them, disorganisation	Responses related to chaos in regulations of primary health care and lack of information update considering proceeding rules	*‘An inextricable, ever-changing set of proceeding rules’; ‘not being informed and updated on time’*
Increased work-, thus stress load and responsibility due to COVID and unavailability of specialist care	Responses related to shot down of specialist care, thus having increased workload and responsibility	*‘Unavailable specialist care’; ‘I felt helpless that hospital and clinic care had actually ceased’*
Fear, worry, unreliable information, uncertainty	Responses related to uncertainty, lack of reliable information and predictability	*‘Uncertainty, daily changing rules, chaos;’ ‘fear, ignorance’*
Panic and concern of patients and to calm and inform them	Responses related to the burden of calming panicking patients and giving them reliable information	*‘Reassuring patients’; ‘the dread that the patients pounded on me’*
Lack of protective equipment	Responses related to not having access to protective equipment when possibly being exposed to the virus	*‘Lack of protective equipment;’ ‘the impossibility of obtaining protective equipment’*
Protecting own health, wearing mask, sanitising	Responses related to anxiety about own safety	*‘protecting my assistant’s and my health;’ ‘take care of the patient while I stay healthy’*
Lack of professional contact and help, incompetence of professionals	Responses related to unavailability of consultation with colleges of other specialties because of increased workload	*‘Lack of both professional and political support;’ ‘unavailable specialist clinics;’ ‘tolerate the incompetence of epidemiologists’*

**Table 3.** Categories of COVID-related personal challenges of GPs with description and example responses.

Category	Description of category	Example response
Lack of personal contact	Responses related to being separated from loved ones and acquaintances	*‘Lack of personal encounter;’ ‘lack of personal communication’*
Increased home workload, organisation, online education	Responses related to pressure at home to manage work, housekeeping, online education of children at the same time	*‘Doing my work and taking care of the children in parallel, mainly studying with my school-age children;’ ‘helping my children learn at home’*
Curfew, travelling restrictions	Responses related to lack of freedom and curfew	*‘My trip abroad had to be cancelled;’ ‘the confinement’*
Opening restrictions	Responses related to difficulties to run errands due to restrictions of opening hours	*‘The time limit of shopping because my wife and I couldn’t shop at the same time’*
Loss of mental balance, need of psychological help	Responses related to mental health problems and needing psychological help	*‘To face my state of mind, my limits, my need for help’; ‘psychic tension’*
Financial problems	Responses related to losing job and facing a financial crisis	*‘The financial deficit due to the loss of side jobs’*
Loss of loved ones	Responses related to mourning passing loved ones	*‘death of my husband’*

**Figure 2.**
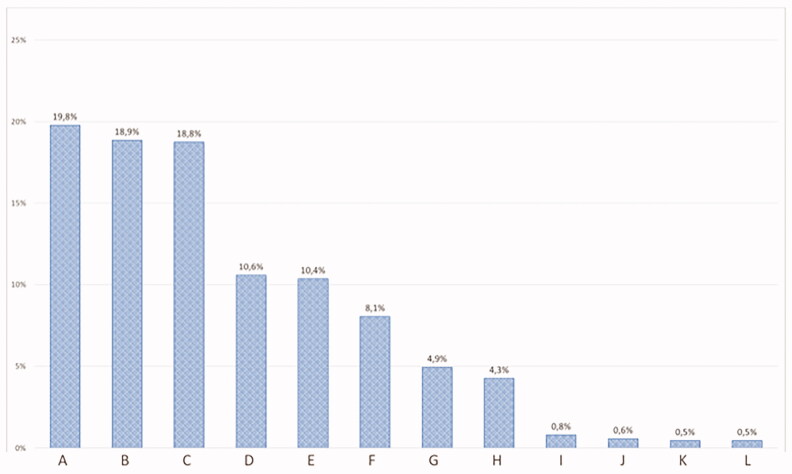
Percentage distribution of recreational activities reported by Hungarian GPs (*n* = 228). (A) Connection with nature, (B) Reading, watching movies, (C) Physical exercise, (D) Meeting friends and acquaintances, (E) Cooking, (F) Praying, meditation, (G) Creative manual activities, DIY, (H) Beautification, cosmetics, (I) Spending time with children, grandchildren, (J) Making and listening to music, (K) Gardening, (L) Training, learning, educational tasks.

To create categories of stress releasing recreational activities, we selected the Mental Health Foundation (UK) ‘How to manage and reduce stress’ booklet as well as the American Counselling Association’s article ‘100 Ways to Reduce Stress: Making the Balancing Act More Manageable’ to base our choices. We offered multiple possibilities for recreation (connection with nature, reading or watching movies, physical exercise, meeting friends and acquaintances, cooking, praying or meditation, creative manual activities and DIY, or beautification and cosmetics) and participants were able to provide their answers on their sources of recreation as well. Their answers were then sorted and counted and presented in Figure 2.

### Statistical analyses

Chi square tests were used in case of the categorical data, two-tailed *t*-test for normally and Kruskal-Wallis test for non-normally distributed continuous variables. Dunn’s pairwise tests with Bonferroni adjustment for multiple comparisons were carried out for the three pairs of groups. Normality of data was assessed using the Kolmogorov-Smirnov test. In our cross-sectional study, we applied step forward likelihood ratio logistic regression analysis to estimate the role of age, sex, place of living, the number of chronic diseases, the number of stressors and the number of days the respondents applied at least 30-min recreation in the exposure to allostatic overload.

We applied 95% confidence intervals (CI). In all cases, a *p* value < 0.05 was considered statistically significant. We used SPSS-24.0 software (SPSS Inc., Armonk, NY, USA).

## Results

### Sociodemographic and COVID-related characteristics

After excluding 13 double fill-outs, we analysed the data of 228 GPs, 155 of whom were females. The youngest doctor was 32 years old while the oldest was 88. We did not find any statistically significant differences in health-related and sociodemographic characteristics according to sex (Table 4).

**Table 4.** Sociodemographic and health-related characteristics of a Hungarian general practitioner sample during the COVID-19 pandemic (*N* = 228).

	Males (*N* = 73)	Females (*N* = 155)
Variables	Mean (SD)	Mean (SD)
Age	56 (12)	57 (10)
	*N* ***(%)***	*N* ***(%)***
Place of living, *n* (%)		
capital	22 (30)	67 (43)
county seat	12 (16)	20 (13)
town	30 (41)	48 (31)
village	9 (12)	20 (13)
Number of chronic diseases participating doctors had		
0	23 (32)	48 (31)
1–2	38 (52)	84 (54)
3–5	12 (16)	20 (13)
5<	0 (0)	3 (2)
Psychiatric disease (yes)		
	1 (1)	5 (3)
Number of medications participating doctors were taking themselves		
0	25 (34)	51 (33)
1–5	40 (55)	93 (60)
6≤	8 (11)	11 (7)

No significant difference was found between the two groups on any of the variables.

We found that 222 colleagues worked during the first wave of the pandemic. Two-thirds of them (155) worked in person in the surgery during the pandemic. They all used mixed – personal, phone calls/video calls and online – possibilities for consultation.

### Sources of stress

According to GPs’ answers on the most challenging aspects of the pandemic and the related quarantine, qualitative data showed that work-related conditions and increased workload were the most challenging for the majority of GPs (Figure 1). Electronic prescription and the use of virtual health service space increased dramatically, causing challenge for less frequent users. Structural changes in delivering care (from personal to online and phone consultation) as well as decreased possibility for outpatient specialty care, stood as the most essential points. Additionally, they dealt with increased responsibility of calming and informing patients while also in fear and uncertainty (Figure 1 and Table 2).

As shown in Figure 1, general practitioners mainly reported professional challenges as most burdening but personal difficulties yield important as well (Table 3).

### Prevalence of allostatic overload and factors associated with it

Allostatic overload with somatic symptoms of distress or impaired social and occupational functioning was experienced by 60% (*N* = 131) of the sample. Female sex (OR: 1.99; CI: 1.06; 3.74, *p* = 0.032) and the increasing number of chronic daily stressors (OR: 1.4; CI: 1.2; 1.6, *p* < 0.001) both associated with increased odds of allostatic overload while each more day with time for recreation associated with 20% lower odds (OR: 0.838; CI: 0.72; 0.97, *p* = 0.020) after adjusting for age, place of living and chronic diseases.

### Recreation and well-being

Two hundred and seventeen (95.2%) out of 228 family physicians reported doing something actively for their health in general. They reported spending at least 30 minutes on recreation an average of 4 days a week. We offered multiple possibilities for recreation to choose from besides individual answers. The median number of different recreation types chosen was 4 (IQR: 3, 5). The most popular forms were connection with nature, reading or watching movies and physical exercise (Figure 2). When grouping family physicians according to the number of days they recreated, we found that being involved in recreation at least 5 days a week associated with lower point scores on symptoms of anxiety, depression, somatisation, and hostility while just 3 days weekly associated with elevated scores on mental and physical well-being (Table 5).

**Table 5.** Mental health parameters of general practitioners according to the number of days they spent at least 30 min for recreation during the week (*N* = 228).

	30 min/0–2 days (*N* = 55)	30 min/3–4 days (*N* = 75)	30 min/5–7 days (*N* = 98)
Anxiety (SQ)	6.00 (3.00;12.00)	6.00 (2.00;8.00)	3.00 (1.00;7.25)*
Depression (SQ)	6.00 (3.00;11.00)	3.00 (2.00;7.00)^§^	3.50 (1.00;7.00)*
Somatisation (SQ)	6.00 (4.00;12.00)	4.00 (2.00;8.00)	3.50 (1.00;7.00)*
Hostility (SQ)	7.00 (2.00;12.00)	5.00 (1.00;10.00)	3.50 (1.00;9.00)*
Mental health well-being (PHS-WB)	4.40 (3.20;4.60)	4.40 (4.00;4.80)^§^	4.40 (4.00;4.80)*
Social well-being (PHS-WB)	4.50 (3.50;4.50)	4.50 (4.00;5.00)	4.50 (3.63;5.00)
Physical well-being (PHS-WB)	3.30 (2.30;4.00)	4.00 (3.33;4.50)^§^	4.33 (3.33;4.66)*
Total well-being (PHS-WB)	3.77 (2.94;4.33)	4.24 (3.80;4.65)^§^	4.26 (3.67;4.71)*

SQ: Kellner Symptom Questionnaire; PHS-WB: Public Health Surveillance Well-being Scale; Medians and (IQRs) can be seen in cells. *Significant difference between 0–2 days and 5–7 days; §: significant difference between 0–2 days and 3–4 days.

## Discussion

### Main findings

We found that 60% of participating Hungarian family physicians suffered from allostatic overload in relation to adverse life events during the first wave of COVID-19 pandemic. Females and those experiencing more stressors in their lives were more vulnerable. Each additional day when time was ensured for 30-min recreation associated with 19% decreased odds of this vulnerability. Elevated mental and physical well-being associated with at least 3 days; lower symptoms of depression, anxiety, somatisation, and hostility, with 5–7 days recreation weekly.

### Strengths and limitations

There is insufficient literature mapping general practitioners’ mental health, but even those few concentrate mostly on negative aspects of it [14]. It is a rarity to find studies on resources to promote well-being which also support the ability to cope and perform under extreme stress circumstances. The strength of our research is to explore distress symptoms (depression, anxiety, hostility, and somatisation) as well as well-being (mental, physical, and social) under an acute stressor (COVID-19) amongst GPs. We defined the association of regular recreation with lower distress levels alongside with higher level of mental and physical well-being.

Our online survey reached an 18% response rate. Since response rates of 70% or higher are considered good, our response rate is low. Compared to other web-based GP surveys [24], however, our response rate did not seem inferior to others with similar constructions. One shortcoming of our data collection was that we could not separate non-respondents who did not receive the invitation (invalid email addresses) from those who did not provide a fill-in. Approximately one-third of the email addresses belonged to the surgery and not the doctor. High workload and administrative workload are main sources of GPs’ nonresponse to surveys. Our results show that the COVID pandemic put extraordinary burden on GPs (increased workload was the fourth most important source of stress). This might increase the possibility of nonresponse, especially when the request arrived at surgery-related email address. Online surveys are less preferred than paper-based among family physicians. Computer illiteracy might be one cause for that. The finding supports that participant GPs found the changes in consultations most challenging. We found that our respondents were slightly younger than the average age of Hungarian general practitioners (57 years in the sample vs 64 years in the total population) and consisted of more female general practitioners (68% vs 53%). Similarly, French and Swiss GP respondents of a web-based survey were younger and contained fewer males than the community-based GP population [24]. COVID-19 related changes in professional and personal life or emotional or psychological discomfort related to this topic could also influence participation. Recreational sources are individual sets of interests, relations, values, and goals developing throughout life, and practising them is advised by experts to prevent ‘corona phobia’ [25]. Although we could predict their role in lowering the odds of allostatic overload, defining a true causal relationship will be achievable by longitudinal research.

### Allostatic overload and the most important factors associated with it in GPs during the first wave of COVID-19

The first wave of the ongoing pandemic shed light on the psychosocial burden health workers faced [26,27]. Job strain, social isolation, fears of stigmatisation and uncertainty about the future added to stress, exhaustion, and depressive mood nurses and doctors had experienced [27]. While most studies focus on those in close contact with COVID-19 patients [20,26,27], quantitative data about the types and levels of COVID-19 related stress among family physicians are scarce, even though they are first contact to most patients. Recent research in a hospital environment has confirmed that job strain and uncertainty about the future were the most common causes of higher levels of stress and depressive mood healthcare workers experienced [28]. Our results are in line with these findings, showing that in primary health care settings changes in working conditions, uncertainty and emotional issues multiplied the burden of the pandemic on them. Females and those who experienced additional stressors simultaneously to the pandemic were at higher risk. Exploring mental ill-health and constituents of GPs’ well-being enhance the knowledge in the field.

### Mental health and well-being of GPs and regularity and forms of recreational activity

According to literature, general practitioners are more depressed than white-collar workers [29] and experience higher patient-related stress than other medical specialists while their self-estimated health and workability is lower [30]. However, the well-being of British general practitioners was comparable to the local population, and GPs above 55 years showed higher hope and optimism than their younger counterparts [31]. Our sample showed comparable levels of mental and social well-being during the COVID-19 pandemic to a community sample [21]; however, anxiety and hostility scored higher, probably referring to the high level of additional professional stress load. Most of the GPs ensured time regularly for recreation. According to our results, higher frequency of weekly recreation associated with higher mental and physical well-being and lower distress symptoms. A recent review article on interventions highlights that besides psychotherapeutic programmes [14], increasing awareness on thoughts, beliefs, self-care, personal health and self-care boundaries improved mental health. Our results strengthen these findings because individually chosen types of recreation were equally able to improve mental health. This is even more important during the burdening time of the pandemic, when besides psychosocial support and a better infrastructure adjustment, leisure time is the second biggest resource following interpersonal connectedness [28].

### Implications for practice

Besides providing eminent care for patients, it is of utmost importance to take conscious care of ourselves. Recreational activity can be easily achieved and is provenly effective in maintaining better mental and physical health and significantly reducing distress symptoms. Actively applying 30 minutes of recreation 5–7 days a week might dramatically improve our ability to succeed.

## Conclusion

Our study demonstrates that Hungarian general practitioners were burdened by the first wave of COVID-19, with 60% of the participating physicians presenting allostatic overload. Professional challenges were most demanding, and females and those experiencing additional life stresses were more vulnerable. Regular recreation associated with elevated mental and physical well-being, lower distress symptoms and lowered odds of AO. Longitudinal research is needed to support our results further.
